# Evaluating the effectiveness of Uganda’s Supranational TB Reference Laboratory quality management system training program

**DOI:** 10.1186/s12960-023-00876-8

**Published:** 2023-11-21

**Authors:** Nakiwala Dorothy, Benjamini Niringiyimana, Wekiya Enock, Ocung Guido, Kabugo Joel, Adam Isa, Anita Katuramu, Orena Beatrice, Christine Nansubuga Korsah, Lillian Kyomugasho, Denis Oola, Kenneth Musisi, Eunjung Kim, Nayeong Yu, Ruth Kaliisa, Yeni Lee, Bounggui Kim, Ssenyonga Ronald, Noah Kiwanuka, Katamba Achilles, Moses L. Joloba

**Affiliations:** 1 National Tuberculosis Reference Laboratory/WHO Supranational Reference Laboratory, Kampala, Uganda; 2 Korea Foundation for International Healthcare (KOFIH), Seoul, Korea; 3 College of Health Science, Makerere University, Kampala, Uganda

**Keywords:** LQMS, SLIPTA, WHO AFRO checklist, NTRL accreditation, Training, Sub-Saharan Africa, SRL

## Abstract

**Background:**

Achieving the targeted organizational goals through effective training can increase employee satisfaction. Since 2015, the Supranational Reference Laboratory Uganda (SRL Uganda) has trained National Tuberculosis Reference Laboratories (NTRLs) from 21 countries in a variety of areas that cover both technical and programmatic aspects pertinent to TB laboratories. The Laboratory Quality Management System (LQMS) under SRL coordinates actions intended to ensure sustained quality of the laboratory services offered by the National TB Reference Laboratories. In order for laboratory results to be helpful in a clinical or public health setting, they must be accurate, reliable, and timely. The LQMS course aims to provide learners with knowledge on how to attain and maintain this quality. Prior to this study, there was hardly any data available on the effectiveness of LQMS trainings provided by SRL Uganda; using Kirkpatrick model, which is popular among researchers for evaluating the efficacy of the training program, this paper seeks to establish the effectiveness of the LQMS training offered by the SRL Uganda.

**Method:**

We evaluated the effectiveness of LQMS training within the Uganda’s SRL network for courses offered during the period 2017 and 2021 for participants from the Southern and East African sub-Saharan region.

**Results:**

In 2017 and 2021, respectively, test results from 10/17 and 9/17 showed overall post-test scores above 80%. Of the 18 labs evaluated, 14 showed improvement; of these, 7 labs were from the Eastern region and the other 7 were from Southern Africa; one facility in this region also maintained its accreditation. In the post-evaluation assessment, attendees of the LQMS course gave feedback of strongly agree and agree variety.

**Conclusion:**

More SRL Uganda network laboratories in the regions achieved a 5-star SLIPTA level rating and among these, 5 NTRLs got ISO 15189:2012 accredited by the end of 2021, while one maintained its accreditation status. This proves that the Laboratory Quality Management System training program was an effective tool in improving the quality of laboratory services, work practices, and processes.

## Background

Training is a useful investment and is one of the most important factors in human resource development [1]. Through training, employers can shape employees’ competencies and develop their potential to perform tasks [2] more efficaciously toward attainment of organizational goals. Effective training can improve employee satisfaction by motivating them and making them realize their potential and career goals [1].

The Uganda National Tuberculosis Reference Laboratory (NTRL) which doubles as a Supranational Reference Laboratory (SRL) since 2013 has immensely expanded the frontiers of its mandate beyond providing external quality assurance and supporting disease surveillance studies in the region; to becoming a training HUB in TB diagnostic programs.

For the last 7 years since 2015, Uganda has provided trainings to the regional NTRLs from 21 countries based in sub-Saharan African on both technical and programmatic areas. The level of trainings is internationally recognized with the SRL-Uganda’s training program being accredited by International Accreditation for Continuous Education and Training (IACET) in 2021. The trainings include development of National Strategic Plans (NSP), preparation of Proficiency Testing (PT) panels, implementation of phenotypic and genotypic Drug Susceptibility Testing (DST), Bioinformatics (Genomics), Biosafety & Biosecurity and implementation of Laboratory Quality Management System (LQMS), Benchmarking visits among others.

The most crucial aspect of medical laboratory testing is the implementation of LQMS that wholly coordinates activities that direct and control laboratories with regard to quality (ISO 15189:2012). The LQMS course offered by SRL-Uganda aims to impart participants with knowledge on how to achieve quality laboratory results with insights of accuracy, reliability, and timeliness to positively impact clinical or public health decisions. The LQMS training is conducted for 10 days (41 contact hours) with methodology of knowledge and skills transfer following didactic sessions. The training materials were developed by SRL-Uganda with reference to ISO 15189:2012 and global laboratory initiative (GLI).

For any training course to show effectiveness, there is need to provide trend analysis of implementation among the trainees enrolled on the program over time to recognize achievement of its intended outcomes [3]. It has been documented that training evaluation is a critical component of analyzing, designing, developing, and implementing an effective training program, creating room to also identify training needs [4–10].

Using Kirkpatrick model, we evaluated the reaction, learning, and results impact of QMS training program within Uganda SRL network participating laboratories based in two African regions (Eastern, and Southern Africa) over two time points (2017 and 2021). Kirkpatrick’s model assesses the effectiveness of training programs at four levels: (1) response of the trainee to the training experience (including training experience); (2) the learner’s learning outcomes and increases in knowledge, skill, and attitude toward the attendance experience (how much attendees learned the content after training). This level usually measured through using a pre-test and post-test; (3) the students’ change in behavior and improvement (whether the learning transferred into practice in the workplace); and (4) results (the ultimate impact of training) [4, 7, 8].

## Methods

### Study design and participants

This was a retrospective cross-sectional study that was conducted at the SRL-Uganda to evaluate the effectiveness of the LQMS offered for the period 2017 and 2021.

The study involved laboratory personnel under the SRL-Uganda network that underwent two LQMS training in 2017 and 2021. The countries included Kenya, Tanzania, Somalia, South Sudan, Burundi, Eritrea, Rwanda, and Somaliland from the Eastern region, and Lesotho, Zimbabwe, Zambia, Malawi, Botswana, Mauritius, Seychelles, Mozambique, and Namibia from the southern region.

The LQMS training entailed the 12 Quality System Essentials (QSE) that included Organization, Personnel, Equipment, Purchase and Inventory, Process control, Information Management, Documents and records, Occurrence Management, Assessment, Process Improvement, Customer service, and Facilities and Safety.

### Measurement variables

We used The Kirkpatrick’s Model that is a framework with four measurable levels designed to evaluate the effectiveness of training programs, i.e., Reaction, Learning, Behavior, and Results [4]. Three out of the four levels of the Kirkpatrick’s model were evaluated under this study as follows:

For Level 1—Reaction; we assessed the general satisfaction and perception of participants about the training courses they had received from SRL Uganda. Data were collected using self-administered evaluation forms at the end of the training course and these were anonymized to ensure confidentiality. The evaluation form consisted of different parameters on which participants were assessed and this includes Relevance of the training to participants’ work, overall rating of the training, overall training objectives/outcomes met, ability to apply the knowledge and whether the training met participants’ expectations. For each question, scores were graded using Likert scale from 1 to 5 (1—strongly disagree, 5—strongly agree).

For Level 2—Learning; participants’ knowledge on the course content was assessed before and after the LQMS training course to determine if the learning objectives had been met. Data on participants’ performance were collected from pre- and post-tests for this evaluation. Satisfactory performance was achieved if a participant scored above 80% in the post-test.

For Level 4—Results; we assessed the outcome and impact of the training using Data derived from the LQMS scores using the WHO AFRO SLIPTA assessment checklist and evidence of record accreditation within the study period.

### Data analysis

We analyzed data using STATA ver 15.0. We performed a two-sample test for proportion. We described the countries by region and sex of the participants. Data were presented in tabular form.

Level 1: Reaction—to determine for each item/question: relevance of the training to participants’ work, overall rating of the training, overall training objectives/outcomes met, ability to apply the knowledge and whether the training met participants’ expectations, we summed up the total number of participants who responded to each category on the Likert scale and we used a bar graph to present the total number of participants who responded to each of the five questions along the categories of the Likert scale.

Level 2: Learning—we compared the proportion of participants who score ≥ 80% between the pre- and post-test scores by region in 2017 and 2021, respectively. We assessed for any significant difference (*p* ≤0.05) in the knowledge of participants between the pre- and post-test scores.

Level 4: Results—we compared the Quality Management System status of the laboratories before and after the LQMS training based on the WHO AFRO SLIPTA assessment results and certificates of international accreditation to ISO 15189:2012.

## Results

A total of 34 participants from 17 countries in sub-Saharan Africa were evaluated for LQMS trainings organized in 2017 and 2021 by Uganda SRL. Of these, 20 participants were from the Southern African region while 14 were from the Eastern African region, we had 20 females and 14 males (Table 1).

**Table 1:** Number of participants that attended the LQMS training in 2017 and 2021 by region and sex

African regions	Sex		Overall
	Male	Female	
Eastern	9	5	14
Southern	11	9	20
Total	20	14	34

Eastern African region (Kenya, Tanzania, Somalia, South Sudan, Burundi, Eritrea, Rwanda and Somaliland), Southern African region (Lesotho, Zimbabwe, Zambia, Malawi, Botswana, Mauritius, Seychelles, Mozambique and Namibia)

An overall post-test score of above 80% was achieved by 10/17 and 9/17 participants in 2017 and 2021, respectively. There was a significant (*p* = 0.001 and < 0.001) improvement in participants’ knowledge over the two trainings. Better performance was observed in the Southern African region in both 2017 and 2021(Table 2).

**Table 2:** Performance scores from the LQMS training in 2017 and 2021 (using the learning level in the Kirkpatrick’s Evaluation Model with an 80% cut off)

African regions	2017		Two proportions *p*-value
	Pre-test	Post-test	
Eastern	0/7	2/7	
Southern	1/10	8/10	
Overall-1	1/17	10/17	0.001

	2021		
	Pre-test	Post-test	
Eastern	0/6	2/6	
Southern	0/11	7/11	
Overall-2	0/17	9/17	< 0.001

**Fig. 1:**
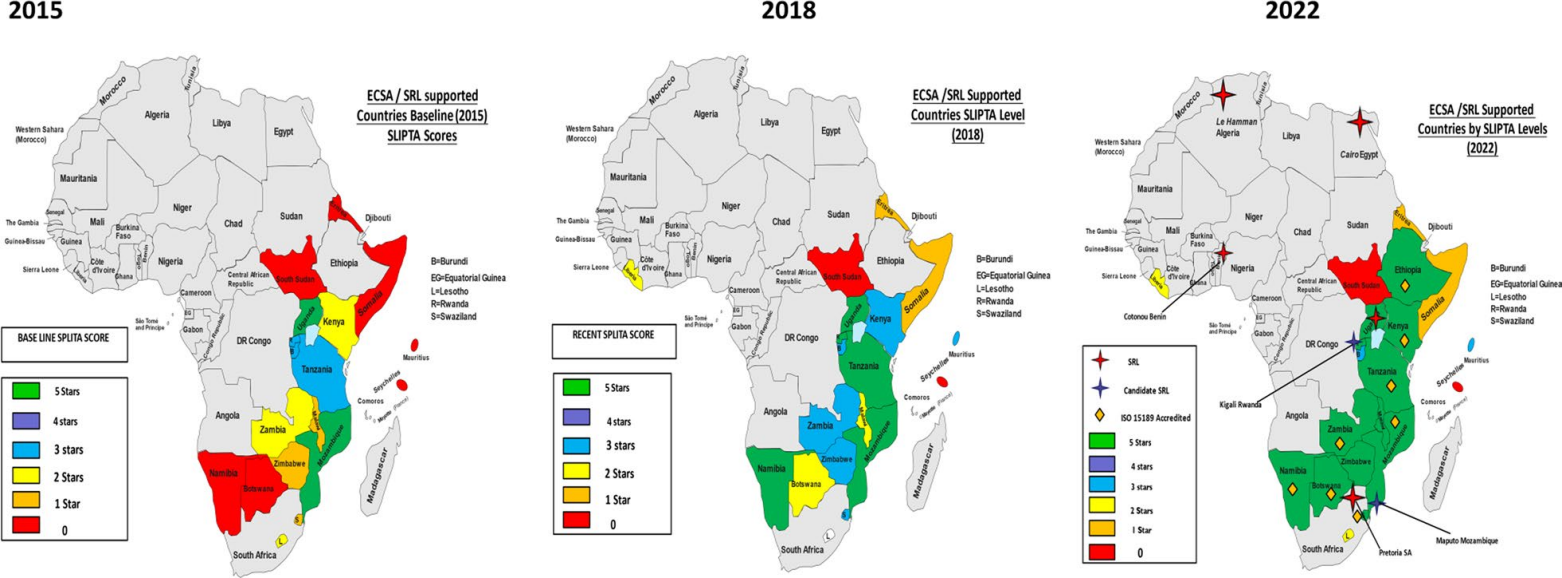
Choropleth map of ECSA/SRL-Uganda supported countries comparison by WHO AFRO SLIPTA level rating at baseline (2015), mid project (2018) and current level (2022), (before and after project intervention) (2015–2022)

Of the 17 country laboratories that were assessed, 14 showed improvement, among these, 7 laboratories were from Eastern African region and 7 from the Southern African region, 1 facility maintain its accreditation status. In terms of WHO AFRO SLIPTA star-level rating, there was a tremendous improvement with more SRL Uganda supported laboratories progressing from star 0, 1, 2, 3, 5 in 2017 to star 1, 2, 3, and ISO 15189:2012 Accreditation (Fig. 1).

In 2015, from baseline data we had 7 laboratories at 0 stars (Red color), 3 labs at 1 star (Orange color), 2 labs at 2 stars (Yellow color), 3 labs at 3 stars (Blue color) and 2 labs at 5 stars (Green color). After the initial LQMS training in 2017, we observed a tremendous improvement where a number of labs moved from star 0 (Red color) to star 3 (Blue color) and 5 (Green color) and eventually 8 laboratories got accredited for ISO 15189:2012 by 2022 post-LQMS training.

**Fig. 2:**
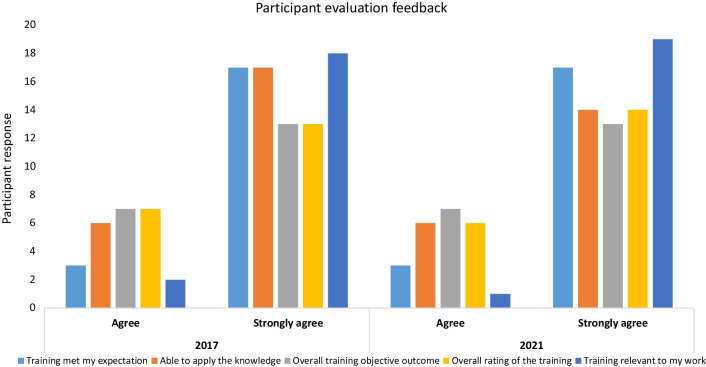
Training evaluation feedback for participants that attended the Laboratory Quality Management System (LQMS) training in 2017 and 2021 as organized by SRL Uganda

Strongly agree and agree were the only evaluation responses from all the participants that attended LQMS training in both 2017 and 2021 as reflected in Fig. 2

## Discussion

In this cross-section study, we evaluated the effectiveness of LQMS training program at Supranational Reference Laboratory Network. The study focused on the three domains of the Kirkpatrick’s program evaluation model that includes reaction, learning, and results.

The findings of the study indicated that the LQMS training program was effective based on the three domains.

As far as reaction is concerned, participants were satisfied with the LQMS training in terms of relevance to their work, training objectives/outcomes, participants’ expectations met and will be able to apply the knowledge gained. These findings are similar to studies conducted by [11], effect of in-service educational courses on human resources’ efficiency from university experts’ point of view suggested that half of the staff believed that the workshop was perfect.

Our study findings showed that participants from two regions had a significant improvement in knowledge over the two LQMS trainings with an overall countries’ laboratories post-test score of 10/17 and 9/17 respectively in 2017 and 2021. The finding further showed a better performance in the Southern Africa region in the 2 years compared to the Eastern region. The results suggest that the training program was one of the effective way in increasing participant knowledge. In line with our study, [12] found that training was effective in increasing the participants’ learning and knowledge in study where they examined the effect of in-service training on cardiopulmonary resuscitation using Kirkpatrick’s model [12].

In this study, we show that 14 (78%) country laboratories significantly improved as per the WHO AFRO SLIPTA assessment and international accreditation (includes maintenance and attainment). Half of the countries’ laboratories were from the Eastern Africa region. These results showed that participants used the knowledge attained to improve practices and processes in their laboratories. It is possible that some of the improvement we observed was due to post-training activities such as technical assistance and regular follow-up supervisory visits, ongoing mentorship, and affordable, cost-effective bench marking visits to countries with similar operating environments, backgrounds, and cultures [13, 14] as well as other special fundings. The extensive Proficiency Testing Program offered by SRL Uganda to regional NTRLs at no cost was crucial in removing a stumbling block toward accreditation.

Further analysis of the results revealed that two labs did not significantly improve while three labs kept their star rating of three stars. High staff turnover, poor knowledge transfer, a limited leadership commitment to quality improvement, and the belief that TB is not a big problem making it a non-priority area worth funding all could have played a role in this, despite being heavily resourced [15] Due to the full-time commitment and participation of LQMS mentored staff, we were able to witness that laboratories in conflict zones with equally limited resources made a significant shift from star 0 to star 1 despite the upheavals. In addition, SRL Uganda is currently providing them continuous technical support as they move closer to ISO 15189:2012 accreditation. The strength of this study is that it included representative participants from East and Southern Africa region and tasked them with the sole responsibility of enhancing the quality of TB diagnostic services. This study used the standardized structured tools to collect data used in evaluating the training program.

The study did not evaluate the third level of the Kirkpatrick model and was limited by availability of published data on all the four Kirkpatrick model levels to compare our findings with findings elsewhere. The training program evaluation was further limited by the ability of participants to reply to the surveys in English with varied clarity, which led to some confusing or shortened responses, which further limited the responses for program evaluation. The choice of the study design limited comparability of our results with laboratories that did not receive LQMS training.

## Conclusion

The Laboratory Quality Management System training program was effective in improving the quality of laboratory work practices and processes. Further research ought to be carried out in assessing the 3rd level (behavior) of Kirkpatrick's model in assessing the impact of LQMS training on improving the quality of laboratory services and to understand the causal relationship and isolate the impact of the training program from other concurrent or subsequent support.

## Data Availability

The datasets used and/or analyzed during the current study are available from the corresponding author on reasonable request.
